# The Out-Patient Problem

**Published:** 1908-10-10

**Authors:** 


### HOSPITAL ADMINISTRATION.

### CONSTRUCTION AND ECONOMICS.

### THE OUT-PATIENT PROBLEM.

### BIRMINGHAM'S SCHEME FOR ITS SOLUTION.

Birmingham is a great city, and has a deservedly high reputation for the acumen and intelligence with which its inhabitants conduct their local affairs. Public opinion in Birmingham is more concentrated than in most cities, and there is usually a commendable desire to reach a solution whenever a new problem presents itself. In The Hospital of April 13, 1907, we gave an account of the steps which were being taken at the General Hospital with the object of preventing out-patient abuse. What is known as a filtration scheme was set up, the effects of which have been to deal with a considerable number of cases by simply giving them first aid and telling them not to attend the hospital again. So far the General Hospital is the only hospital in Birmingham which has adopted this system, but Mr. Neville Chamberlain, who is responsible for the present attempt at solution, states that the first thing to secure is co-operation among hospitals instead of competition. At the present time the hospitals compete with each other for out-patients. They do not like out-patients although they lay themselves out to get as many out-patients to attend as possible, so that they may have striking figures for publication in the annual report. The example of the General Hospital in adopting a filtration scheme is, Mr. Chamberlain believes, however, about to be followed by every hospital in Birmingham. Unless such co-operation is established the General Hospital plan must largely fail, because the people turned away from that institution are able to obtain relief at a neighbouring hospital.

#### The Position in Birmingham.

At Birmingham there are a few provident dispensaries, and an institution known as the General Dispensary which has branches all over the city, with resident medical officers in charge, which has for decades of years attended an increasing number of people. The difficulty the General Hospital is experiencing in pursuing its scheme of filtration is due to the fact that medical men do not like to refuse medical treatment to a case unless they can refer it to some other source where the required relief can be obtained under proper conditions. To effect this it is essential that Birmingham shall be divided into districts, that each district shall have a centre where the patients needing medical relief can obtain it for a sum within their means on a provident basis. It was evidently quite impossible to raise a fund and establish new provident dispensaries all over the city in any case, and the difficulty was immensely increased by the readiness with which free medical relief can be at present obtained from the General Dispensary. Again, the Hospital Saturday Fund has been more successful in Birmingham and has raised more money there in proportion to the population than in any other city. There are also a great number of medical clubs, and all these factors had to be taken into consideration when drafting a scheme for the solution of the present out-patient problem. Mr. Neville Chamberlain has worked hard at the question, and has succeeded in securing the appointment of a Committee for his new scheme by the General Medical Practitioners' Union at their annual meeting. This Committee consists of Drs. Hallwright, White, Neal, G. Bryce, Ensor, Pooler, and Burgess.

#### The Birmingham Scheme in Outline.

It cannot fail to be of interest to summarise in outline the proposals with which this Committee will have to deal. Starting with the conviction that the present state of things in regard to medical relief can only be cured by the gradual education of the people, Mr. Neville Chamberlain and his Committee have determined to endeavour to change the people's habits and thought, so that when they are ill, instead of going to a hospital, they may go to their own doctor first, and only resort to the hospital when they are told to do so by the doctor, should he find their case requires it. Mr. Chamberlain recognises that most of the people referred to are unable to pay the ordinary medical fee, but they can pay something. For this reason it is felt to be well to educate them to pay some regular contribution when they are well, which will entitle them to medical relief when they are ill, and so to acquire the habit of thrift. The first aim of the Committee will be therefore to educate the people, and this can only be accomplished by co-operation. Co-operation must be obtained

(1) Amongst all hospitals, in substitution for the present competition. This great reform, as we have just shown, it is hoped has been practically secured.

(2) To establish the means whereby the people who may be refused relief at the hospitals can get equally good treatment elsewhere. With this in view it has been decided to establish in Birmingham provident dispensaries intended to supplement the existing agencies and especially to relieve the out-patient departments of the hospitals of trivial cases. These dispensaries will be complementary to the hospitals, and the latter will send their trivial cases to the former for treatment.

(3) To set up in addition to the few existing provident dispensaries, with the object of preventing an increase of the present agencies for the distribution of medical relief, provident dispensary centres in each of the district buildings at present managed by the General Dispensary. The Dispensary Committee will place the buildings at the disposal of Mr. Chamberlain's Committee for this purpose, and subsequently the necessary clerical staff. The Provident Dispensary Committee will therefore commence with the advantage of being able to establish a provident dispensary in whatever part of the city of Birmingham they consider desirable.

(4) To secure the co-operation of the medical profession, and with this in view it has been decided to appoint as many general practitioners as possible on the staff of the provident dispensary. The appointment of the Committee on the 29th ult. by the General Medical Practitioners' Union guarantees, we presume, the support of the medical profession for Mr. Chamberlain's scheme.

### The Medical Clubs to be Superseded.

Mr. Neville Chamberlain is of opinion that this scheme will supersede Medical Clubs altogether, and so far as he can understand the feeling of practitioners generally they would not be sorry to see the Medical Clubs done away with. The scheme offers two advantages compared with the Medical Club:

(1) A man who has not a large practice cannot afford to keep a large stock of expensive medicines, and so the scheme provides that medicines should be supplied by the General Dispensary, which is able to keep a large stock of medicines always available. A further advantage will be that by this plan the cost of the drugs can be reduced, because of the large quantity that will be used.

(2) The practitioners will be saved the collection of their fees, as the whole of the collection will be done by the provident dispensary.

Again the medical profession is to be amply and liberally represented on the Committee of Management. The City of Birmingham Aid Society and the Hospital Saturday Fund will also be represented. It is proposed to have a number of local committees to supervise the working of the districts and to keep each division in constant touch with what is going on in other districts. The financial basis of the scheme rests upon a plan whereby it is agreed that seventy per cent. of the fees shall be given to the medical staff, twenty per cent. to the General Dispensary, and ten per cent. to the clerical staff employed.

We shall watch the working of this scheme with great interest. It is no doubt true, as Mr. Chamberlain contends, that the majority of hospital patients at present have the choice of two things. Either they have to pay more than they can afford, or they take charity and pay nothing for their medical treatment. This scheme will afford an opportunity for the exercise of thrift and independence on the part of this class of people now that the medical profession have agreed to extend to it their assistance and co-operation. Mr. Chamberlain properly claims that if this scheme is adopted and thoroughly worked, Birmingham should lead the way in a reform of medical relief which is likely to be extensively adopted in other cities and towns throughout the country.

